# Reduction of Dislocations of the Femur by Manipulation

**Published:** 1858-03

**Authors:** Frank Hastings Hamilton


ART. II.—*Reduction of Dislocations of the Femur by Manipulation. Fragments of the Literature of this method.* By FRANK HASTINGS HAMILTON, M. D.

(Continued from Page 520.)

Kluge directs in case of a dislocation of the hip upwards and backwards, that the patient should be placed upon his back and his body well secured by towels; an assistant must now flex the leg upon the thigh, and the thigh upon the body, at the same moment rotating the knee inwards and abducting the thigh. The surgeon will then lift the thigh and give it a slight pull

---

or twist. *Chelius' Surgery, vol.* ii, *p.* 241; from *Sich, G. R., Dissect. de lux. Femoris. Berol.*, 1823, *p.* 26.

Wattmann places his patient also on his back, and making use of straps with one or two assistants, in case the dislocaticn is upwards and backwards, he either draws the thigh in its own axis downwards, and by the aid of the thigh strap outwards and downwards, or he seizes the limb at the knee and ankle, and then slowly lifts it forwards, until the thigh is at right angles with the body. If now the thigh is found to rotate outwards by its own inclination, and in a manner which the hands of the surgeon cannot resist, the movement of the head of the femur over the socket is declared, and it only remains to let the limb fall gradually downwards until it lies close to the other upon the bed. Thus the reduction is accomplished. These movements are varied in the other dislocations of the hip to suit the action of the muscles. *Chelius, op. cit.*, from *Wattmann. Vienna*, 1826.

According to Rust's plan the patient is also laid upon his back; then bending the thigh upon the body, and while extension and abduction is made by an assistant, through the action of the muscles, "it springs into its socket with an audible noise, and without the employment of any considerable degree of extension being requisite." *Chelins. Note by South*, from *Richter*, p, 706. 1828.

"Colombat proposes a mode of reduction which he has always successfully employed without assistance and without pain to the patient. The patient stands upright upon the undislocated limb, his chest bent forwards and resting on a table or high couch, with his hands grasping the opposite sides of the table or couch, to keep his body immovable during the operation. The surgeon places bimself behiud the patient, on the inside of the dislocated limb, if the dislocation be forwards; but on the outside, if it be backwards. He puts first one hand above the tarsus, to bend the leg upon the dislocated thigh; the other hand, which lies behind the knee, is employed to make gradual pressure from above downwards, for the convenience of extending the muscles. With the first hand he imparts to the limb gentle motions from right to left, and from before to behind, in order to overcome the opposition of the muscles of the thigh, and to render the head movable, which then moves from the place in which it is, and enters the socket with a noise." *Chelius. Note by South, vol.* ii, *p.* 241. 1830.

"The following interesting case of compound dislocation of the femur was communicated to me by my former pupil, Dr. Walker, of Charlestown, near Boston, in North America:

"CASE LXIII. The patient, while driving a wagon laden with many tons weight of manure, seated upon the "spire," received a blow which threw him upon the ground, and, as he describes, resting upon his elbows and knees. In this position the wagon passed over the posterior part of his pelvis and right thigh, forcing the head of the femur out of the acetabulum forwards upon the groin, lacerating the soft parts and pressing the head through the integuments. This was, in fact, the state of things when I was called to him. The man was extremely muscular, and having been drinking freely, though not to intoxication, his muscles were greatly agitated. He was restless, and complained of great suffering: the diagnosis was extremely easy, every symptom of dislocation on the pubes being present. By the concurrent advice of my friend Dr. Shurtleff, in whose service the patient had received the injury, I proceeded to make an immediate effort at reduction, in the manner directed in your inestimable treatise, excepting that, having no pulleys, we availed ourselves of manual assistance. The efforts at reduction produced great suffering, but did not succeed in removing the head of the bone from its new situation, notwithstanding the extension was made by several strong men, and in a variety of directions. Foiled in my first attempt, I placed my patient in bed, had him largely bled, and gave him a powerful dose of laudanum. Early the next morning I visited him, and found him calm, free from pain, and evidently under the influence of opium, and in every way well fitted for a further attempt at reduction, which I determined upon attempting with the use of the pulleys. My friend, Dr. William Ingalls, who was now present, requested he might be first allowed to make an effort without the pulleys, and, to my astonishment, he succeeded in replacing the head of the bone in the acetabulum in the following manner: By his direction an assistant, taking the ankle of the dislocated limb in his right hand, and placing his left in the ham, bent the leg at right angles upon the thigh, and the thigh upon the pelvis, then lifting with a power little more than sufficient to elevate the whole limb, he carried it to its greatest state of abduction, at the same time rotating the femur inwards while Dr. Ingalls passed his thumbs through the wound, and pressing upon the head of the femur directed it towards the acetabulum. At this moment he directed the limb to be forced towards its fellow, by which the reduction was effected with the greatest ease and elegance. Whether the same manner would succeed in other luxations upon the pubes I know not, but here I

state facts. I regret to add, that extensive suppuration proved fatal to our patient at the end of about three weeks." *A Treatise on Dislocations and Fractures of the Joints. By Sir Astley Cooper. Edited by Bransby Cooper. Amer. Ed.* 1851. (The English edition was published in 1842.) p. 119.

"*Dislocation of the Femur on the Dorsum Ilii, reducible without Pulleys, or any other Mechanical Power. Three Cases.* An Essay read before the Monroe County Medical Society, at its annual Meeting, in the city of Rochester, on the 8th May, 1850. By W. W. REID, M. D.

"DR. FLINT,

"Dear Sir:—The following paper was read before the Monroe Co. Med. Society, at its annual meeting, in the city of Rochester, on the 8th of May, 1850. For reasons, which are obvious, and *some, which are not so obvious*, I am induced to offer it for publication in your Journal. If you deem it admissible, and worthy, will you give it an insertion in your next number.

"Respectfully and truly yours,

"Rochester, June 24th, 1851. W. W. REID.

"GENTLEMEN: I propose to show that dislocation of the femur on the dorsum ilii, may be reduced without pulleys, without Jarvis' adjuster, without Fanhestock's twisted ropes, without an assistant, in less time, and with far less pain, than by any mechanical means whatever, simply by the hand and strength of the operator alone.

"The announcement of a proposition so novel, so ultra, and contradictory to the teachings of all standard writers on surgery for the last hundred years, exposes me, I am aware, to the sneers of some, to the pity of others, and to the charge of rashness, by all, and requires that I make good my statement by undoubted and substantial proof.

"The subject matter of this paper has been written, but withheld from the public and profession, several years, principally for two reasons:

"*First.* The theory and practice here recommended are so diametrically opposed to all our highest surgical authorities, whether among the living or the dead, that I have shrunk from the obloquy and opprobium that are apt to attach to an innovator upon long established opinions, dogmas, and practices, especially when held and taught by men in our profession of profound science, and practical skill.

"*Second.* I had to wait some four or five years for an opportunity to put to the test this mode of reducing a luxation of the hip-joint, before a case presented itself in my own practice. In the spring of 1844, the first opportunity offered, but as 'one swallow does not make a summer,' I was still unwilling to venture before the profession, although so far as one case could establish a principle, this one did so, as we shall see directly. During the past year, (1849,) two other cases have fallen into my hands, and have rendered what was before certain to my own mind, 'doubly sure.'"

Dr. Reid then proceeds to describe the "mental process, the observations and experiments" by which he arrived at his conclusions; after which, he adds:

"The conviction was so strong in my mind that this method was certain and practicable, that I no more doubted it then than I do now, after having demonstrated it in three several instances, two of which were within the last year. And so impatient was I to put my theory to the test, that I believe I almost wished every day (wickedly perhaps) that some one would dislocate his hip, and give me an opportunity to reduce it.

"I was aware that Professor Nathan Smith, of New Haven, had, in his day, taught in his lectures, a somewhat similar method,—perhaps the same; but none of his pupils, whom I had ever met, could describe either his method or the rationale of it. I had seen, too, his memoirs, published by his son, Professor N. R. Smith, of Baltimore, but he confesses that he did not recollect the teachings of his own father, and that he, the elder Smith, had left no notes or records of his doctrines or practice. Dr. N. R. Smith, however, proceeds to give what seems to him the probable doctrines inculcated by his father, and gives directions for reducing dislocations of the hip, with drawings illustrative of his method. But it is apparent, that, when he wrote his book and gave these directions and illustrations, he had never reduced a hip by his method. For his directions require impossibilities, and his illustrations are mere fancy; no such thing in nature can exist. For to *abduct* a thigh dislocated on the *dorsum* of the *ilium*, before flexing it on the pelvis, or to *abduct* and *flex* at the same time, as he directs, is absolutely impossible, without rupturing the obturator externus—and to rupture this, in order to obtain flexure, would require the power of many men;* *but to flex the leg first on the thigh—then adduct the thigh, carrying it even over the sound*

---

* Note.—Dr. Reid has scarcely done justice to the method of procedure described by Dr. Nathan R. Smith, when he understands him to say, that in carrying the limb up it is to be abducted forcibly, or more than the limb is capable of being abducted without doing violence to any of the muscles; since Dr. Smith says distinctly, as I have quoted at p. 510, that the thigh is to be abdueted only "slightly," before it is flexed upon the body; and not until the thigh is flexed "freely" upon the body is the abduction to be completed.

*one and at the same time, flex the thigh on the pelvis, carrying the knee over and upwards by a kind of semi-circular sweep,* is a very different and a very easy thing."

Dr. Reid proceeds to relate three cases in which he has succeeded by this method in reducing dislocations of the hip, and to describe other experiments made in connection with Prof. Moore, of Rochester, with a view of determining what constituted the real obstacles to the reduction. From all of which he deduces the following conclusions:

"1. The chief impediment in the reduction of dislocations, is the indirect action of the muscles that are put upon the *stretch* by the mal-position of the dislocated bone, and not by the *contraction* of the muscles that are shortened.

"2. That muscles are capable of so little extension, without hazard of rupture, beyond their normal length, that no attempt should be made to stretch them further, in order to reduce a dislocation, if it can possibly be avoided.

"3. The general rule for reducing all luxations should be, that the limb or bone should be carried, moved, flexed or drawn, in that direction which will relax the distended muscles.

"4. Dislocation of the hip on the *dorsum ilii*, an accident so serious to the patient, and so formidable to all surgeons, is reduced with the greatest ease, in a few minutes, without much pain, without an assistant, without pullies, without 'Jarvis' adjuster,' or any other mechanical means, simply by flexing the leg upon the thigh, carrying the thigh over the sound one, upward over the pelvis, as high as the umbilicus, and then by *abducting* and rotating it."—*Buf. Med. Jour., vol.* vii, *p.* 129 *et seq.* (*Aug.*, 1851.)

(To be Continued.)

